# Metabolome and transcriptome analyses of the molecular mechanisms of flower color mutation in tobacco

**DOI:** 10.1186/s12864-020-07028-5

**Published:** 2020-09-07

**Authors:** Fangchan Jiao, Lu Zhao, Xingfu Wu, Zhongbang Song, Yongping Li

**Affiliations:** 1 Yunnan Academy of Tobacco Agricultural Sciences, Kunming, 650021 Yunnan China; 2 National Center for Tobacco Gene Engineering, Kunming, 650021 Yunnan China

**Keywords:** Tobacco, Flower coloration, Anthocyanin, Mutation, Gene expression

## Abstract

**Background:**

Anthocyanins determinate the flower color of many plants. Tobacco is a model plant for studying the molecular regulation of flower coloration. We investigated the mechanism underlying flower coloration in tobacco by profiling flavonoid metabolites,expression of anthocyanin biosynthetic structural genes and their regulator genes in the pink-flowered tobacco cultivar Yunyan 87 and white-flowered Yunyan 87 mutant.

**Result:**

Significant down-accumulation of anthocyanins, including cyanidin 3-O-glucoside, cyanin, cyanidin 3-O-rutinoside, pelargonidin 3-O-beta-D-glucoside, cyanidin O-syringic acid, pelargonin, and pelargonidin 3-O-malonylhexoside (log_2_ fold change < − 10), endowed the flower color mutation in Yunyan 87 mutant. Transcriptome analysis showed that the coordinately down-regulated anthocyanin biosynthetic genes including chalcone isomerase, naringenin 3-dioxygenase, dihydroflavonol 4-reductase and UDP-glucose:flavonoid 3-O-glucosyltransferase played critical roles in suppressing the formation of the aforesaid anthocyanins. Several genes encoding MYB and bHLH transcription factors were also found down-regulated, and probably the reason for the suppression of structural genes.

**Conclusion:**

This is the first study of tobacco flower coloration combining metabolome and transcriptome analyses, and the results shed a light on the systematic regulation mechanisms of flower coloration in tobacco. The obtained information will aid in developing strategies to modify flower color through genetic transformation.

## Background

Flower color is a key trait for ornamental plants, and flower coloration has been one of the hotspots in biological studies [1, 2]. Anthocyanins, carotenoids and betalains are the main pigments in plants [3]. Anthocyanins confer all orange, pink, red, purple, blue and blue-black flower colors [4, 5]. Carotenoids confer yellow color while betalains existing exclusively in Caryophyllales, are responsible for the yellow, orange, red and purple colors. In previous studies, anthocyanin contents were regarded as the major factors endowing the flower color as pink or light red in transgenic tobacco [6–10]. The anthocyanin biosynthetic pathways have been characterized extensively in higher plants such as *Arabidopsis thaliana* [11], *Medicago truncatula* [12], *Fragaria ananasa* [13], *Vaccinium myrtillus* [14], *Trifolium repens* [15], *Antirrhinum majus* [16], *Vitis vinifera* [17, 18], *Daucus carota* [19], and *Zea mays* [20]. The conserved structural genes including chalcone synthase (*CHS*), chalcone isomerase (*CHI*), flavanone 3-hydroxylase (*F3H*), dihydroflavonol reductase (*DFR*), anthocyanidin synthase (*ANS*), and UDP-glucose:flavonoid 3-o-glucosyltransferase (*UFGT*) involved in anthocyanin biosynthetic pathway have been identified [3, 5, 21], but the regulatory mechanisms vary across plant species [22]. The anthocyanidin are glycolysed into anthocyanin by *UFGT* [23]. Flavonoid production has been limited by the inhibition of one step in the biosynthetic pathway in flowers of several plant species, including chrysanthemum, cyclamen, tobacco, gerbera, carnation, gentian, rose, lisianthus, petunia, and torenia [24, 25]. In addition, the anthocyanin biosynthesis is regulated by key transcription factors belonging to the families of *R2R3 MYB*, *bHLH* and *WD40*, interacting together to form a *MYB-bHLH-WD40* (MBW) complex [8, 26–29]. MYB transcription factors are able to induce anthocyanin accumulation in plants [26, 30–35]. While in some cases, some *MYBs* require co-expression of specific *bHLHs* to efficiently induce the anthocyanin biosynthesis [36, 37]. Tobacco (*Nicotiana tabacum*) is an important model plant for studying the molecular mechanisms of flower coloration. Unfortunately, the previous studies of flower coloration in tobacco are limited and mainly focused on the control of one of the core anthocyanin biosynthetic genes or the regulatory factors such as MYB transcription factors [7–10, 24, 25, 36]. Hence, a clear insight into the molecular mechanism underlying the tobacco flower coloration is still lacking. Recently, metabolomics and transcriptomics have facilitated identification of molecules and key genes involved color formation in plants [38–42]. Herein, we profiled the changes in metabolome and transcriptome between pink and white flower petals of tobacco in order to pinpoint key genes controlling anthocyanin composition in the flowers of Yunyan 87 and Yunyan 87 mutant.

## Results

### Metabolome profiling of the petal samples of pink-flowered and white-flowered tobacco

**Fig. 1:**
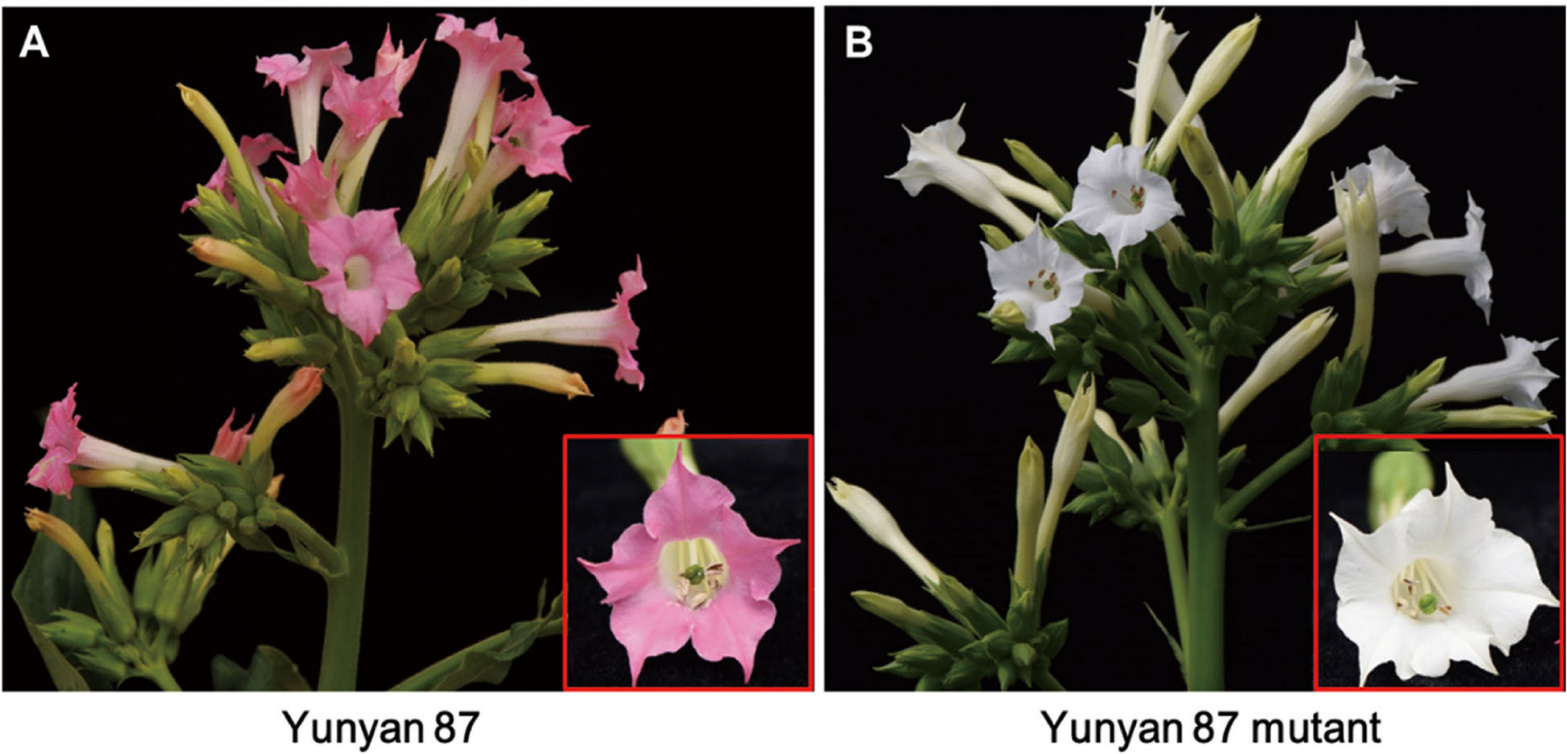
The phenotypes of (**a**) wild type of tobacco cultivar Yunyan 87 and (**b**) its mutant type Yunyan 87 mutant. Y87: Yunyan 87 (pink flower); Y87W: Yunyan 87 mutant (white flower)

Fresh flowers with the pink and/or white petals were collected from tobacco cultivar Yunyan 87 and Yunyan 87 mutant, respectively (Fig. 1a, b). Flower samples were analyzed for metabolite concentrations between pink flowers of Yunyan 87 (Y87) and white flowers of Yunyan 87 mutant (Y87W).

**Fig. 2:**
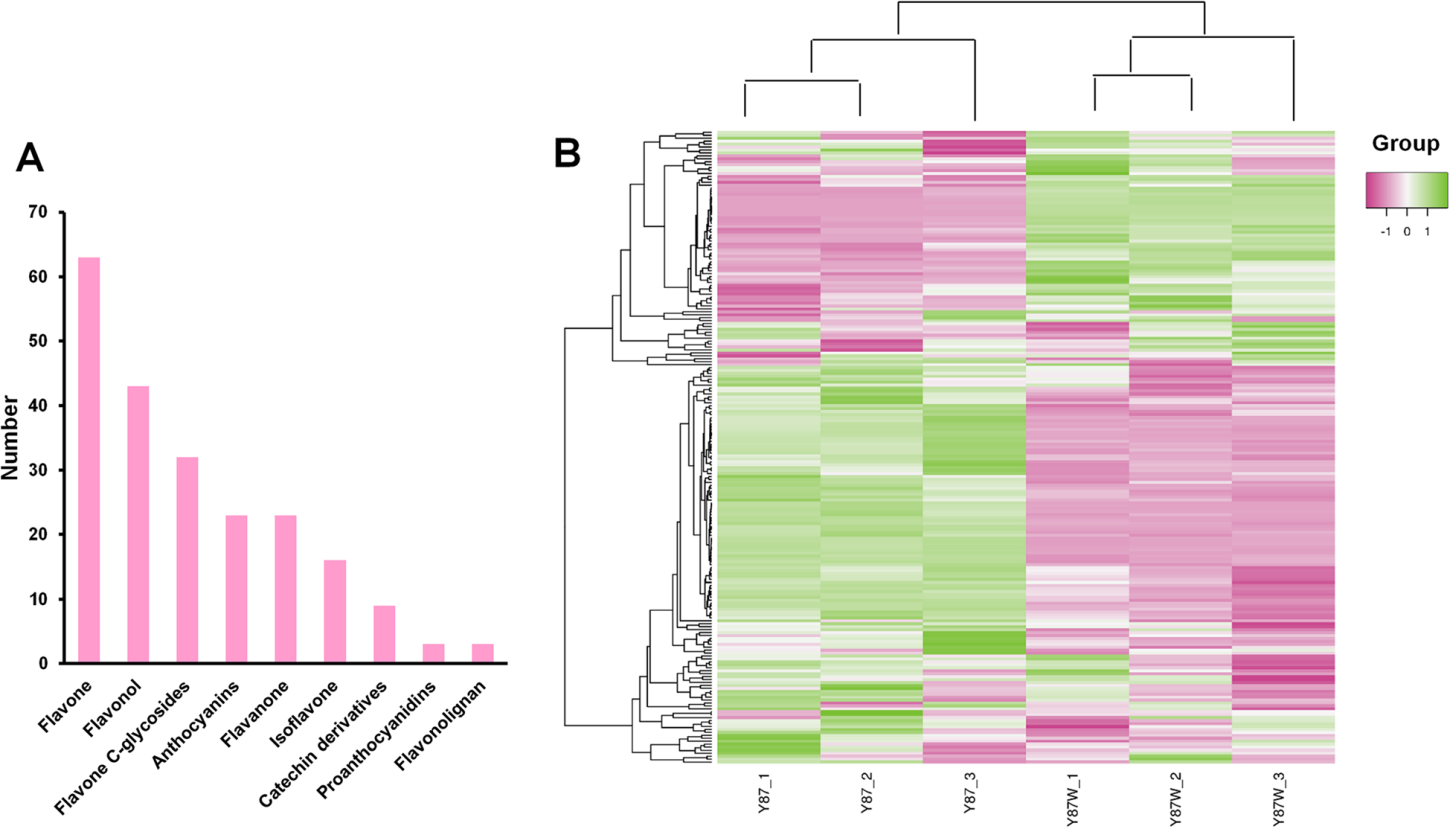
Metabolome profiling of the petal samples of pink-flowered and white-flowered tobacco. **a** Classification of the 215 detected metabolites into nine classes; **b** Heatmap clustering showing correlation between the pink and white flower samples based on 215 metabolite profiles. Y87: Yunyan 87 (pink flower); Y87W: Yunyan 87 mutant (white flower). Color scale from pink to green in the heatmap represents the normalized metabolite contents using Row Z-score

Since anthocyanin content is determinant for flower color change in tobacco [6–10], we profiled the metabolome of the petal samples of Yunyan 87 and Yunyan 87 mutant using the flavonoid-targeted metabolomics approach [42]. Each sample had three replicates, so in total six samples were analyzed. We detected in the tobacco flower a total of 215 compounds, which could be grouped into nine classes including flavone, flavonol, flavone C-glycosides, anthocyanins, flavanone, isoflavone, catechin derivatives, proanthocyanidins, flavonolignan (Fig. 2a; Table S1). A hierarchical heatmap clustering analysis of the petal samples of Yunyan 87 and Yunyan 87 mutant was performed based on the metabolite quantification. All of the biological replicates were clustered together which indicated that the quality of the generated metabolome data was high (Fig. 2b). Interestingly, the pink flower samples (Y87) and the white flower samples (Y87W) were clearly separated. The result indicated that distinct flavonoid profiles in Y87 and Y87W samples. In consistence, the metabolites were also clustered into two main groups demonstrating the opposite accumulation patterns between the pink-colored and white-colored flower samples (Fig. 2b).

### Identification of the differentially accumulated metabolites between pink and white flowers in tobacco

**Fig. 3:**
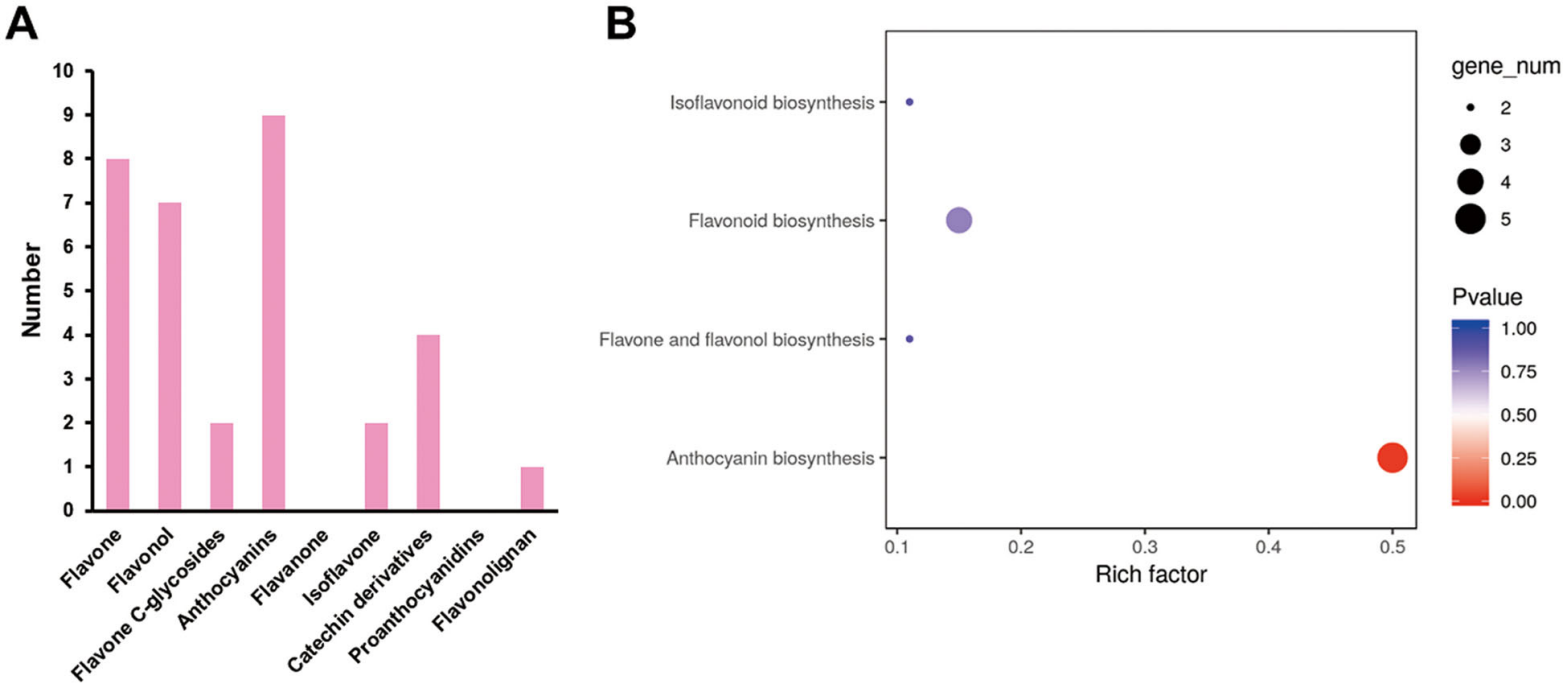
Identification and functional characterization of differentially accumulated metabolites (DAMs) between pink and white flowers in tobacco. **a** The numerous of significantly differentially accumulated metabolites between the pink and white flower samples; **b** KEGG enrichment analysis of the DAMs between the pink and white flower samples based on the method of over-representation analysis (ORA)

The differentially accumulated metabolites (DAMs) in petal samples between Yunyan 87 and Yunyan 87 mutant (Y87 vs. Y87W) were determined based on the variable importance in projection (VIP) ≥ 1 and fold change ≥2 or fold change ≤0.5 [43]. There were 33 DAMs showing significantly different accumulation between the compared samples (Fig. 3a; Table S2). The most enriched KEGG term among the DAMs detected for the compared samples was anthocyanin biosynthesis, based on the method of over-representation analysis (ORA) (Fig. 3b).

Interestingly, all the differentially accumulated anthocyanins were down-accumulated in white flower samples, including the extremely significantly down-accumulated (log_2_ fold change < − 10) anthocyanins (cyanidin 3-O-glucoside, cyanin, cyanidin 3-O-rutinoside, pelargonidin 3-O-beta-D-glucoside, cyanidin O-syringic acid, pelargonin, and pelargonidin 3-O-malonylhexoside) (Table 1). This result indicates that the anthocyanin branch was blocked in the white mutant phenotype.

**Table 1:** Differentially accumulated anthocyanins between pink and white flowers in tobacco. Values represent the ion intensity (concentration) of the metabolites

Metabolite code	Metabolite name	Y87 (ion intensity)	Y87W (ion intensity)	Log_2_ fold change	VIP
pmb0550	Cyanidin 3-O-glucoside (Kuromanin)	4,060,000	9.00	−18.8	3.93
pme1777	Cyanidin 3,5-O-diglucoside (Cyanin)	2,780,000	9.00	−18.2	3.87
pme1773	Cyanidin 3-O-rutinoside (Keracyanin)	2,720,000	9.00	−18.2	3.87
pme3392	Pelargonidin 3-O-beta-D-glucoside (Callistephin chloride)	1,510,000	9.00	−17.4	3.78
pmb2957	Cyanidin O-syringic acid	879,000	9.00	−16.6	3.69
pme1793	Pelargonin	66,400	9.00	−12.8	3.25
pmb0554	Pelargonidin 3-O-malonylhexoside	48,300	9.00	−12.4	3.19
pme1397	Pelargonidin	393,000	110,000	−1.84	1.23
pmb0558	Delphinidin O-malonyl-malonylhexoside	44,100	16,900	−1.38	1.06

### Analysis of differentially expressed genes between pink and white-colored tobacco flowers using RNA-sequencing

We synthesized six cDNA libraries from flowers collected from Yunyan 87 and Yunyan 87 mutant plants and generated transcriptome RNA-sequencing (RNA-seq) data in Yunyan 87 and its mutant phenotype Yunyan 87 mutant. The RNA-seq yielded a total of 46.99 Gb clean data with 91.82% of bases scoring Q30 and above (Table 2).

**Table 2:** Overview of the transcriptome sequencing dataset and quality check

Samples	clean_reads	clean_bases	error_rate	Q20	Q30	GC (%)
Y87_1	50,822,512	7,623,376,800	0.35	97.145	92.6	42.99
Y87_2	46,783,110	7,017,466,500	0.37	96.955	91.975	42.855
Y87_3	51,680,598	7,752,089,700	0.345	97.225	92.6	42.875
Y87W_1	54,330,202	8,149,530,300	0.355	97.135	92.31	42.945
Y87W_2	56,821,124	8,523,168,600	0.375	96.875	91.825	42.97
Y87W_3	52,801,194	7,920,179,100	0.365	96.955	92.035	42.985

**Fig. 4:**
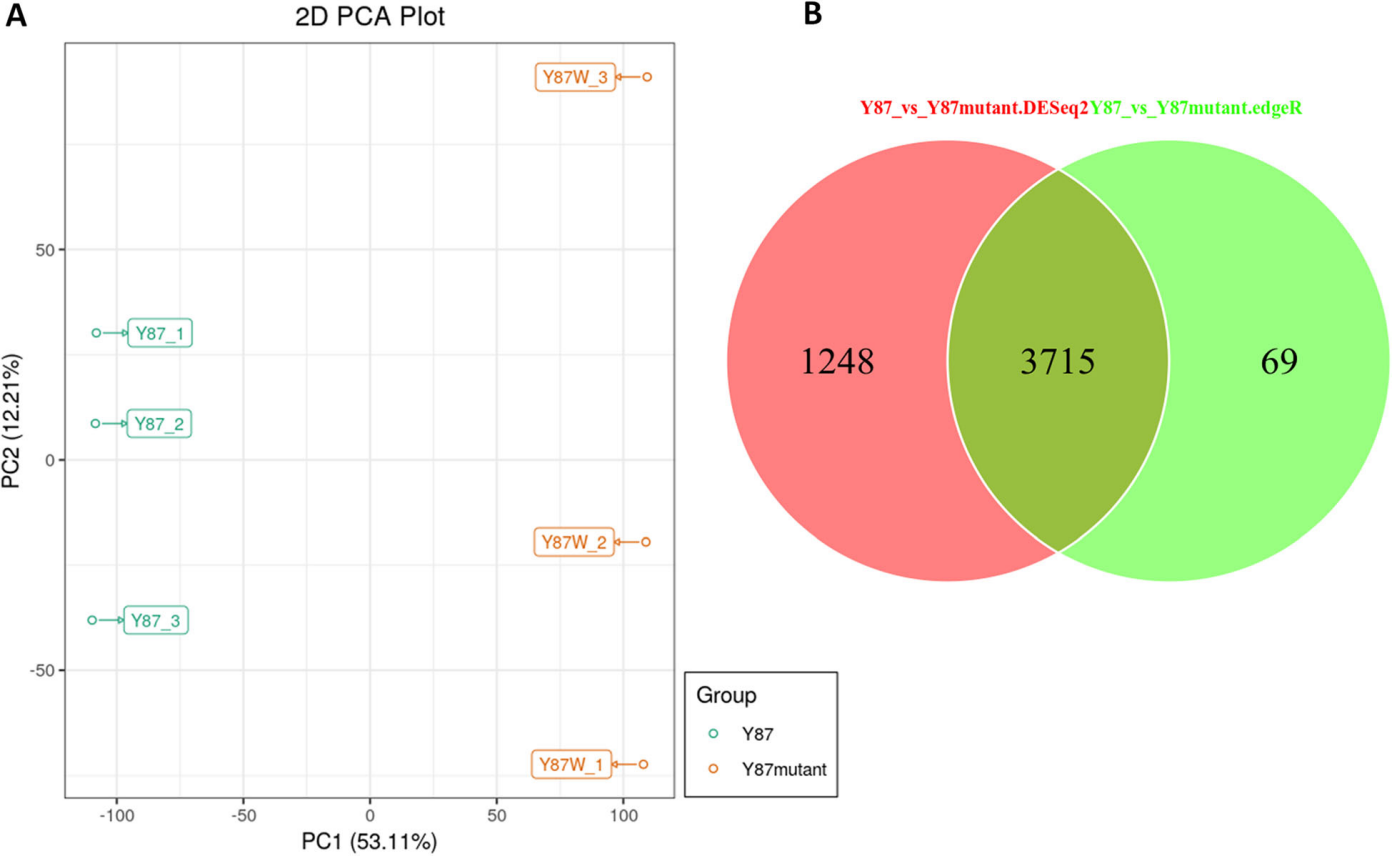
Gene expression of pink and white flowers in tobacco. **a** Principal component analysis based on FPKM data; **b** Venn diagram showing the shared and specific number of DEGs identified by each package. Y87: Yunyan 87 (pink flower); Y87W: Yunyan 87 mutant (white flower)

Of the total clean reads, 83.04 to 85.01% were mapped in proper pairs with the *Nicotiana tabacum* reference genome [44] (Table S3). Principal component analysis (PCA) of the samples based on fragments per kilobase of exon model per million reads mapped (FPKM) clearly separated the two flower sample types, implying that the differentially accumulated metabolites between the two phenotypes are regulated by differential expressed genes (Fig. 4a).

To identify the differentially expressed genes (DEGs) between Y87 and Y87W, the raw standardized count of assembled unigenes were imputed in DESeq and edgeR packages. Only DEGs commonly detected by both packages were used in this study. After comparison, we obtained a total of 3715 DEGs, including 3045 up-regulated and 670 down-regulated genes in the tobacco flowers (Fig. 4b). To further validate the results of the DEGs, eight differentially expressed genes were selected (Table S4) and their expression levels from flowers of the Y87 (pink) and Y87W (white) were analyzed using qRT-PCR. The strongly correlation between the qRT-PCR results and the RNA-seq data (*R*^2^ = 0.8994, Figure S1) confirmed the RNA-seq data obtained in this study are highly reliable.

### Major transcription factors were differentially regulated between pink and white flowers in tobacco

Previous study demonstrated that *MYB* alone, co-expression of *MYB* and *bHLH*, and formation of the *MYB-bHLH-WD40* complex were all sufficient to induce anthocyanin accumulation in plants [8, 29–31, 37]. Given the down-regulated anthocyanin accumulation in white flowers (Table 1), we focused on the most significantly down-regulated transcription factors *MYB*, *bHLH* and *WD40* (log_2_ fold change < − 4). Expression fold change of these TFs showed that three *MYB* genes (*MYB113*, *MYB3*, and *MYB PHL11*) and two *bHLH* genes (*bHLH162*; *gene14770* and *gene27472*) were strikingly down-regulated in white flowers of Yunyan 87 mutant (Table 3; Table S5). In other words, the aforesaid *MYB* genes and *bHLH* genes were significantly up-regulated in pink flower of Yunyan 87. The results indicated that inhibition of the *MYB* genes (*MYB113*, *MYB3*, and *MYB PHL11*) and *bHLH* genes (*bHLH162*; *gene14770* and *gene27472*) may contribute to manipulating the changes in flower color from pink to white in tobacco cultivar Yunyan 87.

**Table 3:** Key transcription factors involved in the flower color change from pink to white in tobacco

Transcription factors	Gene ID	Log_2_ fold change	Gene description
*MYB*	*gene54221*	−9.277	*MYB113*
	*gene14314*	−6.073	*MYB3*
	*gene31475*	−4.868	*MYB PHL11*
*bHLH*	*gene14770*	−5.740	*bHLH162*
	*gene27472*	−4.071	*bHLH162*

### Modulation of anthocyanin biosynthesis and accumulation in tobacco flowers

**Fig. 5:**
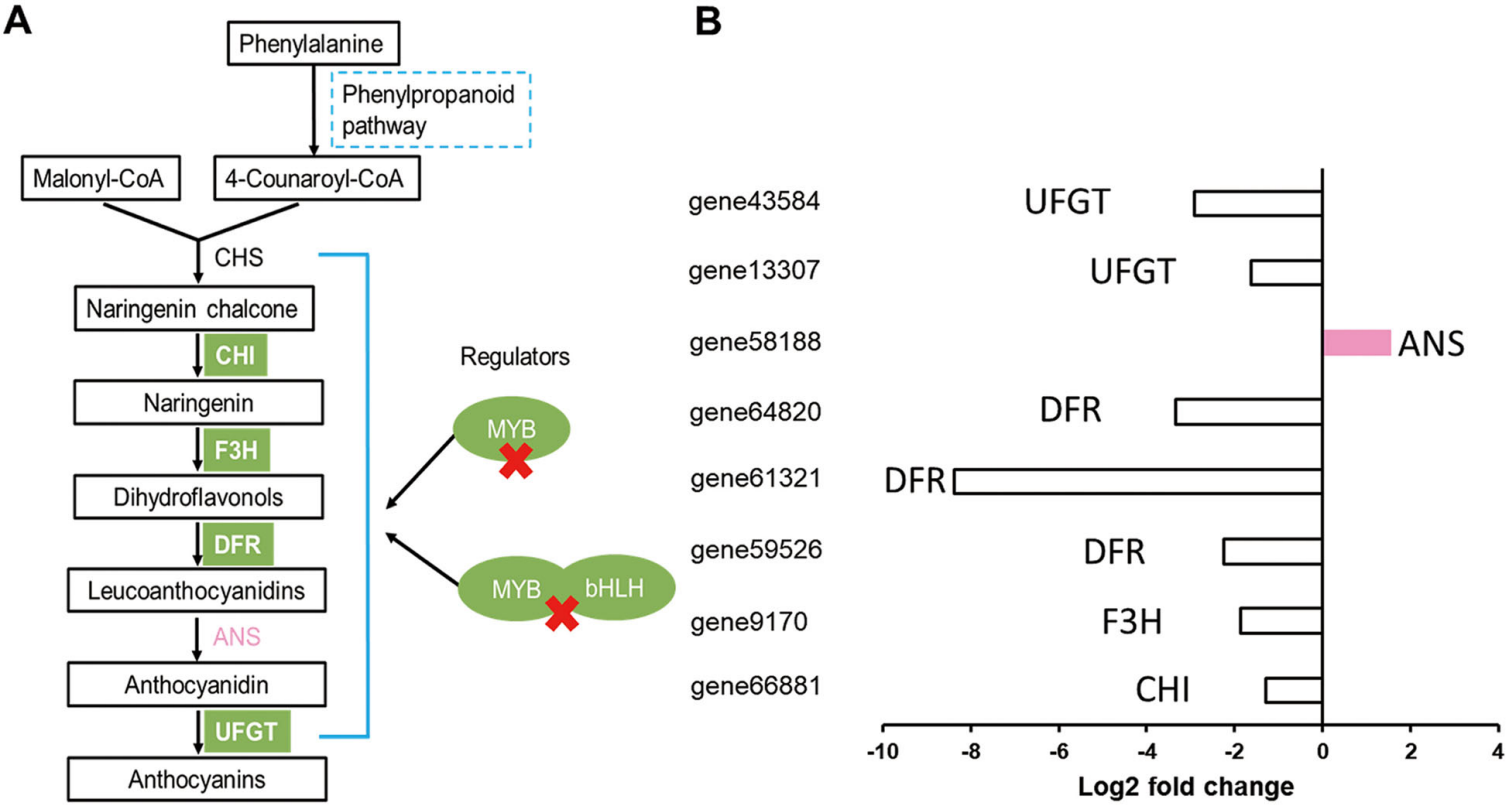
A schematic model of the proposed mechanism underlying the flower color change from pink to white in Yunyan 87 vs. Yunyan 87 mutant. **a** Reconstruction of the anthocyanins biosynthetic pathway with the differentially expressed structural genes and their regulators; **b** The histogram displays the expression levels of the genes (FPKM value). The bars in white and pink colors represent the down- and up-regulated structural genes, respectively. Chalcone synthase (CHS), chalcone isomerase (CHI), flavanone 3-hydroxylase (F3H), Dihydroflavonol 4-reductase (DFR), anthocyanidin synthase (ANS), and UDP-flavonoid:glucosyl transferase (UFGT)

Flower color is mainly determined by the composition and concentrations of the plant’s pigments [1]. In this study, four key genes including *CHI* (*gene66881*), *F3H* (*gene9170*), *DFRs* (*gene59526*, *gene61321*, *gene64820*), and *UFGTs* (*gene13307*, *gene43584*) involved in the anthocyanin biosynthesis were significantly down-regulated, which is consistent with the low anthocyanin contents in Yunyan 87 mutant (Fig. 5a, b). Surprisingly, the enzyme directly involved in anthocyanin biosynthesis, (anthocyanidin synthase; ANS) showed a higher level in Yunyan 87 mutant (Fig. 5b), which contradicts the down-accumulation of anthocyanins. In addition, our study demonstrated that the *MYB* genes (*MYB113*, *MYB3*, and *MYB PHL11*) and *bHLH* genes (*bHLH162*) were significantly down-regulated in white flowers of Yunyan 87 mutant. Taken together, our results suggest that the coordinately down-regulations of *CHI* (*gene66881*), *F3H* (*gene9170*), *DFRs* (*gene59526*, *gene61321*, *gene64820*), and *UFGTs* (*gene13307*, *gene43584*) plays critical roles in suppressing the formation of the anthocyanins including cyanidin 3-O-glucoside, cyanin, cyanidin 3-O-rutinoside, pelargonidin 3-O-beta-D-glucoside, cyanidin O-syringic acid, pelargonin, and pelargonidin 3-O-malonylhexoside (log_2_ fold change < − 10), endowing the changes in flower color from pink to white in tobacco.

## Discussion

The flower color is an important trait in many plants and flower coloration has been one of the hotspots in biological studies [1, 2, 44]. Although color formation is well studied in plants, there are species-specific peculiarities of pigment regulation. For example, some plants only harbor betalains while other harbor anthocyanins as pigments. Moreover, the numbers of structural genes involved in the biosynthetic pathways of these pigments varied considerably across species. Finally, various mechanisms could lead to different colorations such as competition between pathways, mutations in structural genes or regulatory genes, activity of microRNAs, etc. Therefore, studying color formation in a species or new genetic materials can reveal specific genes and specific mechanism of regulation, which will generate new knowledge in this field. In this study, we observed two distinct flower phenotypes in tobacco cultivar Yunyan 87 and Yunyan 87 mutant. The wild phenotype (Yunyan 87) exhibits pink-colored petals, while its mutant phenotype (Yunyan 87 mutant) presents white-colored petals (Fig. 1a, b). Therefore, the tobacco cultivar Yunyan 87 and its mutant12 are desired materials to investigate the systematic molecular mechanisms of flower color mutation in tobacco.

Flavonoids are the major molecules involved in plant pigmentation [37]. There are mainly six groups of flavonoids in plant tissues, including anthocyanins, flavan-3-ols (catechins and proanthocyanidins), flavanonols, flavonols, flavones and phenolic acid [38]. The previous studies on tobacco coloration process were limited to very few flavonoids metabolites such as anthocyanins, flavonols, etc. [7, 45, 46]. In this study, using the flavonoid-targeted metabolomics approach, we investigated the changes in 215 metabolites between the pink flowers of Yunyan 87 and white flowers of Yunyan 87 mutant (Fig. 2a; Table S2), aiming at providing a more comprehensive landscape of the metabolites involved in the flower coloration in tobacco. Analysis of the differentially accumulated metabolites between the two phenotypes revealed that the significantly decreased anthocyanins are the main metabolites modulating the color change of tobacco flowers from pink to white. Anthocyanins comprise a class of water-soluble pigments in plants which contribute to the color of flowers, fruits, stems and leaves [47, 48]*. Previous studies highlighted that a*nthocyanin contents were regarded as the major factors endowing the flower color as pink or light red in transgenic tobacco [6–10]. However, they did not identify the specific anthocyanins responsible for the color alteration of tobacco petals from pink to white. In this study, we found that cyanidin 3-O-glucoside, cyanin, cyanidin 3-O-rutinoside, pelargonidin 3-O-beta-D-glucoside, cyanidin O-syringic acid, pelargonin, and pelargonidin 3-O-malonylhexoside were all extremely significantly down-accumulated (log_2_ fold change < − 10) in white-flowered tobacco in comparison with the pink-flowered tobacco and may be regarded as the major anthocyanins contributing to the color change from pink to white in tobacco.

By manipulating the structural genes of anthocyanin biosynthetic pathway or their regulatory genes, flower colors of several species have been modified [3, 6–10]. We revealed that most of the structural genes (*CHI*, *F3H*, *DFR*, and *UFGT*) involved in the anthocyanin biosynthetic pathway were significantly down-regulated in the white flowers, with the exception of *ANS* gene (Fig. 5b). The up-regulation of *ANS* gene might be the result of feedback regulation [49]. DFR plays a key role in the formation of anthocyanins, directly determines the development of pink or white color in tobacco flowers [50]. Interestingly, our study reveals that *DFR* genes (*gene61321, gene64820, and gene 59,526*) are the most significantly down-regulated genes, which seem to be one of the important candidate genes in determining the flower color in tobacco cultivar Yunyan87. The *UFGT* genes (*gene43584* and *gene13307*) were strictly inhibited in white flowers. It has been reported that the down-regulation of *McDFR* in apple fruit reduced the expression levels of some structural genes (*F3H*, *F3’H*, *DFR*, *ANS* and *UFGT*), while the *CHS* and *CHI* genes were up-regulated [51]. It indicates that the altered expression of *DFR* also affected the expression of other anthocyanin biosynthetic genes. In this study, it is likely that the significant down-regulation of *DFR* genes led to the concomitant decreases in the expression levels of *CHI*, *F3H*, *DFR*, and *UFGT* genes.

MYB alone, co-expression of MYB and bHLH, and formation of the MYB-bHLH-WD40 complex were all sufficient to induce anthocyanin accumulation in plants [8, 22, 29–31, 49]. In tobacco, the regulatory effects of MYB transcription factors (such as *PamMybA.1, PamMybA.3* and *PamMybA.5*) resulted in different accumulation patterns of anthocyanins [8]. Ectopic expression of maize bHLH transcription factor Lc enhanced anthocyanin concentrations in many plants including tobacco [7]. In this study, three *MYB* genes (*MYB113*, *MYB3*, and *MYB PHL11*) and two *bHLH* genes (*bHLH162*; *gene14770* and *gene27472*) were strikingly down-regulated in white flowers of Yunyan 87 mutant (Table 3; Table S5). The down-regulated *MYB* and *bHLH* transcription factors will not activate the expression of structural genes involved in anthocyanin biosynthetic pathway, leading to decreased anthocyanin concentrations. This indicates that the altered flower color in Yunyan 87 mutant might be attributed to the inhibition of *MYB* genes (*MYB113*, *MYB3*, and *MYB PHL11*) and bHLH genes (*bHLH162*; *gene14770* and *gene27472*). However, further studies are required to identify the spontaneously mutated gene(s) in Yunyan 87 mutant, and evaluate how the mutations can cause the reductions in the expression levels of these transcription factors and then the structural genes. This will decipher the systematic regulation mechanisms of the flower coloration in tobacco and benefit the genetic engineering of flower modification in other plants.

## Conclusion

We combined metabolome and transcriptome data to decipher the molecular mechanisms underlying flower coloration in tobacco. By comparing pink and white colored flowers, our data showed that strong down-regulation of anthocyanin biosynthetic structural genes correlated with the significant reduction of anthocyanins in the white flower as compared to the pink samples. Several transcription factors mainly MYB and bHLH were predicted to regulated the anthocyanin biosynthetic structural genes. Collectively, this study offers candidate genes for functional characterization and for manipulation of flower color in tobacco.

## Methods

### Plant materials

The Yunyan 87 mutant, discovered in the field, is a natural mutant of the pink-flowered tobacco cultivar Yunyan 87. The plant materials are available at Yunnan Academy of Tobacco Agricultural Sciences, China. The formal identification of the plant materials was undertaken by the corresponding author of this article (Professor Yongping Li). No voucher specimen of this material has been deposited in a publicly available herbarium. The plants of tobacco Yunyan 87 and Yunyan 87 mutant were grown under controlled conditions at Yanhe, Yuxi, China. The day and night growth temperatures were 28 °C and 25 °C, respectively. During flowering stages, the flowers showed two distinct petal colorations in Yunyan 87 (wild phenotype, pink) and Yunyan 87 mutant (mutant phenotype, white) (Fig. 1). The fresh flowers with pink petals from tobacco cultivar Yunyan 87 and white petals from Yunyan 87 mutant were harvested and named as Y87 and Y87W, respectively. Each Y87 or Y87W sample, consisted of 20 flowers, was frozen in liquid nitrogen and stored at − 80 °C until further use. Three biological replicates (20 individual flowers/replicate) were analyzed for Y87 and Y87W.

### Metabolic profiling

The sample preparation and metabolite analysis were performed according to the methods as previously described by Yuan et al. [43]. The flower samples collected from Y87 and Y87W were crushed to powder and subjected to LC-MS analysis [52].

Quality control (QC) analysis was conducted before the data analysis. PLS-DA analysis was applied to calculate the corresponding variable importance in projection (VIP) value. When the VIP ≥ 1, and fold change ≥2 or fold change ≤0.5, the metabolites were considered as differentially changed metabolites. Heatmap clustering analysis was performed in the R software (www.r-project.org).

### RNA extraction, library preparation, and sequencing

For transcriptome sequencing, six libraries representing the collected petal samples of Y87 and Y87W (three replicates of each) were constructed. Total RNAs were extracted using TRIzol reagent (TaKaRa, China). RNA contamination and RNA integrity number (RIN) were monitored on 1% agarose gels and the Agilent 2100 Bioanalyzer system (Agilent Technologies, CA, USA), respectively. A total amount of 3 μg RNA per sample was used as input material for construction of pair-end (PE) sequencing libraries. Following manufacturer’s instructions, the libraries were generated using NEBNext® UltraTM RNA Library Prep Kit for Illumina® (NEB, USA), and then added index codes to attribute sequences in each sample. According to the manufacturer’s recommendations of TruSeq PE Cluster Kit v3-cBot-HS (Illumina), the clustering of the index-coded samples was performed on a cBot Cluster Generation System. Following the libraries were sequenced by paired-end sequencing on Illumina Hiseq platform.

### Assembled transcriptome data of Illumina HiSeq sequencing

The raw paired-end reads were cleaned through removing adaptor sequences, poly-N, and low quality sequences. The FastQC program (http://www.bioinformatics.babraham.ac.uk/projects/fastqc/) was used to trim the adaptor sequences and low quality sequences (i.e., the percentage of bases of quality value≤5 exceeds 50% in the read). Meanwhile, short sequences (< 50 bp) were also removed using a custom Perl program. The clean data with high quality were applied to the downstream analyses.

Reference genome and gene model annotation files were downloaded from ftp://ftp.sgn.cornell.edu/genomes/Nicotiana_tabacum/edwards_et_al_2017/ [53]. Index of the reference genome was built and paired-end clean reads were aligned to the reference genome using Hisat2 [54]. The transcriptome data of pink-flowered Yunyan 87 and white-flowered Yunyan 87 mutant have been deposited to national center for biotechnology information (NCBI) sequence read archive (SRA) under accession number PRJNA590063 (https://www.ncbi.nlm.nih.gov/Traces/study/?acc=PRJNA590063)

### Differential expression analysis

The reads numbers mapped to each gene were counted using featureCounts v1.5.0-p3 [55]. Then, calculating the expected number of fragments per kilobase of exon model per million reads mapped (FPKM) of each gene based on the length of each gene and reads count mapped to the gene. DEGs of two groups of colored samples were identified using the DESeq R package (v1.18.0) [56] and edgeR package (v 3.24.3). The threshold *p*-value in multiple tests to judge the significance of gene expression difference was based on false discovery rate (FDR) method. When FDR ≤ 0.05 and FPKM values showing at least 2-fold difference among samples, the gene was considered as significant DEG [57]. DEGs commonly detected by both packages were used in this study.

### Validation of gene expression using qRT-PCR

RNA-seq samples (in triplicate) were used for quantitative real time-PCR (qRT-PCR) to test the dependability of the transcriptome results following descriptions of Dossa et al. [58]. Primer pairs of eight selected genes were designed using the Primer Premier 5.0 [59] (Table S4). Data are presented as relative transcript level based on the 2^-∆∆Ct^ method [60]. We estimated the *Pearson* correlation coefficient between the gene expression profiles in the qRT-PCR and RNA-seq in R version 3.6.3.

### GO and KEGG enrichment analysis of DEGs

The GOseq R package [61] was used to analysis of Gene Ontology (GO) enrichment with DEGs. The adjusted *P*-value of significantly substantiated GO terms was less than 0.05. The KEGG pathways enriched with DEGs (FDR < 0.05) were detected using KOBAS 2.0 software [62] based on the method of over-representation analysis (ORA).

## Supplementary information

**Additional file 1: Table S1.** List, characteristics and concentration (ion intensity) of the metabolites detected in pink-colored and white-colored tobacco flowers.

**Additional file 2: Table S2.** List, characteristics and concentration (ion intensity) of the differentially accumulated metabolites between pink-colored and white-colored tobacco flowers.

**Additional file 3: Table S3.** Overview of the transcriptome sequencing dataset and mapping statistics in tobacco flowers collected from Yunyan 87 (Y87) and Yunyan 87 mutant (Y87W).

**Additional file 4: Table S4.** The primer sequences of genes used for quantitative real-time PCR.

**Additional file 5: Table S5.** Expression fold change of up- and down-regulated transcription factors MYB, bHLH and WD40.

**Additional file 6: Figure S1.** qRT-PCR results of 8 selected genes (left) and correlation between transcriptome data and real time PCR results (right).

## Data Availability

RNA-seq data is available at the SRA database in National Center of Biotechnology Information with the accession number PRJNA590063.

## Abbreviations

*CHI*: Chalcone isomerase
*F3H*: Naringenin 3-dioxygenase
*DFR*: Dihydroflavonol 4-reductase
*UFGT*: UDP-glucose:flavonoid 3-O-glucosyltransferase
*CHS*: Chalcone synthase
*ANS*: Anthocyanidin synthase
MBW: *MYB-bHLH-WD40*
LIT: Linear ion trap
QQQ: Triple quadrupole
Q TRAP: Quadrupole-linear ion trap mass spectrometer
MRM: Multiple reaction monitoring
DP: De-clustering potential
CE: Collision energy
VIP: Variable importance in projection
PLS-DA: Partial least squares-discriminant analysis
DAMs: Differentially accumulated metabolites
DEGs: Differentially expressed genes
