# Supplementary figures and images for: Metabolome and transcriptome analyses of the molecular mechanisms of flower color mutation in tobacco
Source: BMC Genomics. 2020 Sep 7;21:611. doi: 10.1186/s12864-020-07028-5 (PMC7487631; doi:10.1186/s12864-020-07028-5)

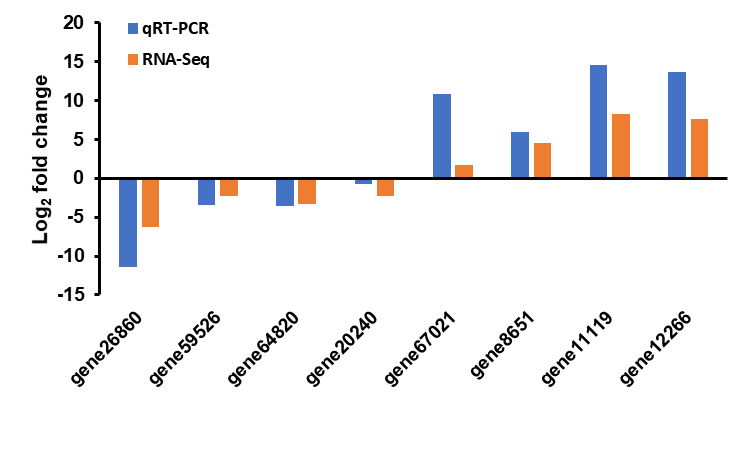

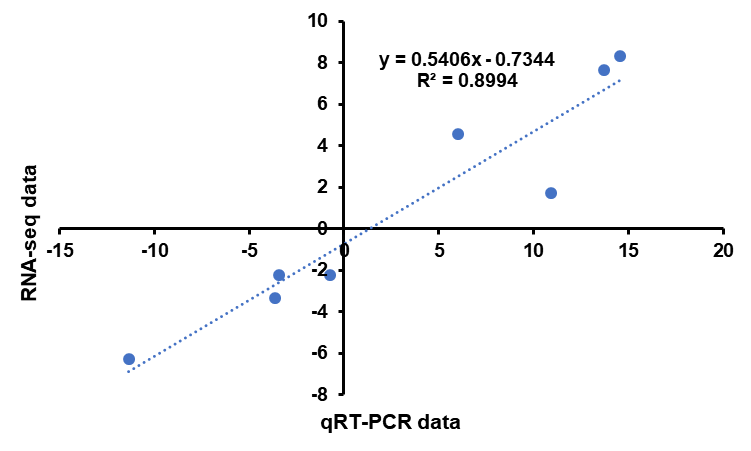

Supplement: Supplementary file 6 — Additional file 6: Figure S1. qRT-PCR results of 8 selected genes (left) and correlation between transcriptome data and real time PCR results (right). [file 12864_2020_7028_MOESM6_ESM.docx]
